# Cystic Glioblastoma: A Mimicker of Infection? A Case Report and Literature Review

**DOI:** 10.1155/2023/7348188

**Published:** 2023-01-17

**Authors:** Ram Prakash Thirugnanasambandam, Gaudy Massiel Vanegas Silva, Alexander H. Wu, Charles J. Kim, Emanuela Cimpeanu, Jeremy Minkowitz, Charles Yanping Shao, Edwin Chiu

**Affiliations:** ^1^Department of Internal Medicine, SUNY Downstate Medical Center, Brooklyn, NY, USA; ^2^Department of Hematology and Oncology, SUNY Downstate Medical Center, Brooklyn, NY, USA; ^3^Department of Pathology, SUNY Downstate Medical Center, Brooklyn, NY, USA; ^4^Department of Pathology, Kings County Hospital Center, Brooklyn, NY, USA

## Abstract

Glioblastoma multiforme (GBM) is the most frequent malignant and aggressive type of glioma. Most cases of GBM present as a single solitary solid tumor; however, there are rare instances in which it may present as a cystic lesion. Here, we report an even rarer case of GBM presenting as bilateral multicystic lesions, mimicking infectious etiology. Our case highlights the importance of identifying clinical features of cystic GBM to ensure early diagnosis and treatment. A literature review was conducted in PubMed, looking at the common characteristics and treatment options for cystic GBM.

## 1. Introduction

Of all the primary tumors of the brain, glioblastoma multiforme (GBM) is the most common malignant and aggressive type [1]. It is a tumor known to arise from astrocytes and is classified as a grade IV astrocytoma. Neuroepithelial tissue tumors account for 30% of all adult primary brain and central nervous system (CNS) tumors by the site. Of these, GBM accounts for half (15%), with an annual incidence rate of 3% [2]. Glioblastoma is usually presented as a single solitary solid tumor; however, there are rare cases in which it may present as a cystic lesion. We report a case of a patient presenting with bilateral multicystic glioblastoma.

## 2. Case Summary

**Figure 1:**
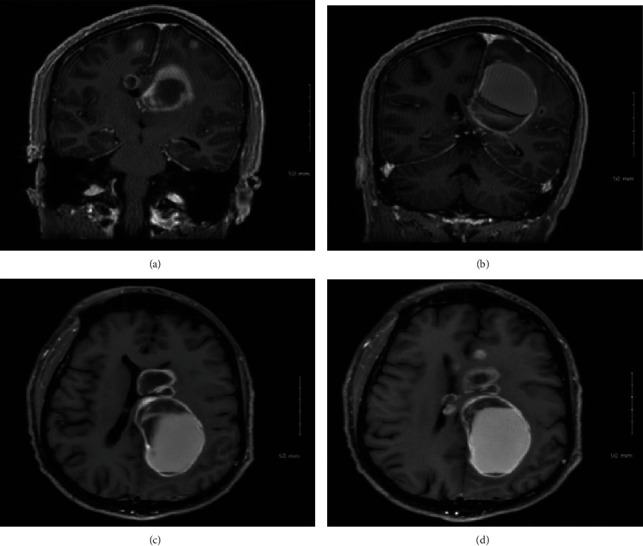
MRI images of the patient's brain. (a, b) T1 postcontrast coronal sequences of the brain showing multiple cystic lesions of varying sizes in the cerebral hemispheres. (c, d) T1 postcontrast axial sequences of the brain showing multiple cystic lesions of varying sizes in the cerebral hemispheres.

The patient is a 40-year-old Haitian man who presented with progressive right-sided weakness, dysesthesias, and headaches. He denies any symptoms of dizziness, vision changes, nausea, vomiting, or neck stiffness. His neurological exam showed upper motor neuron pattern weakness (4 out of 5) in the right upper and lower extremities, increased sensitivity to temperature in his right upper and lower extremities, and gait abnormalities in his right lower extremity. All reflexes were intact and no cranial nerve abnormalities were detected. Computed tomography (CT) scan of the head without contrast showed widespread cystic lesions in the cerebral region of the brain. MRI brain showed numerous bilateral intraparenchymal cystic enhancing lesions, some of which demonstrated internal hemorrhagic components (Figure 1). The largest cystic lesion was in the left paramedian frontoparietal lobe measuring 5.0 × 3.4 × 4.0 cm. It also demonstrated a significant mass effect leading to distortion of the body of the corpus callosum and the body of the left lateral ventricle. Another lesion was found in the left caudate nucleus head region measuring 2.1 × 3.0 × 1.6 cm. A left basal ganglia lesion posterior to the left caudate nucleus lesion had a prominent rim of hemorrhagic products. The septum pellucidum was shifted approximately 5 mm to the right at the level of the frontal horns of the lateral ventricles. On the right, one of the lesions was centered in the white matter of the right superior frontal gyrus measuring 1.8 × 1.5 × 1.6 cm. CT chest and abdomen did not show any primary or metastatic masses. Syphilis serology was nonreactive, and the Quantiferon test was reactive. The patient had no history of latent tuberculosis (LTBI) therapy. Cerebrospinal fluid (CSF) analysis showed a cell count of 10 WBC per mm^3^, protein 69 g/L, and glucose 70 mmol/L. CSF cytology was significant for atypical cells, raising concern for malignancy. Other infectious workup including venereal disease research laboratory (VDRL), routine blood cultures, acid-fast bacillus (AFB), and serum Cryptococcus antigen was negative. Human immune deficiency (HIV) virus, galactomannan, Fungitell, and toxoplasmosis serology were negative.

The patient experienced worsening right lower extremity weakness three days after his admission. His hemiparesis had progressed to a full right lower extremity paralysis. CT head without contrast demonstrated hyperdense material layered within a cystic structure, likely representing blood products. The fundoscopic examination did not report evidence of retinal or subretinal parasites. The patient was started on dexamethasone 4 mg every 6 hours for vasogenic edema, which slightly improved his right-sided hemiparesis and lower extremity paralysis. Despite cysticercosis antibodies being tested negative, the patient was also initiated on albendazole, praziquantel, and levetiracetam for the empiric treatment of neurocysticercosis and seizure prophylaxis. Due to high suspicion of brain tuberculoma, the patient was treated for latent tuberculosis.

**Figure 2:**
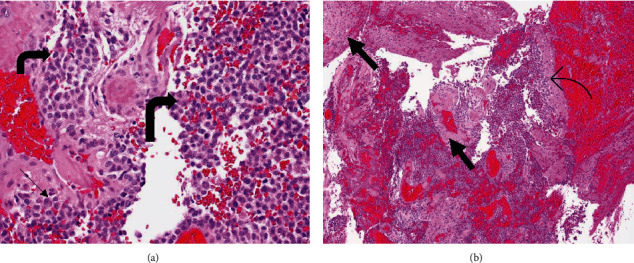
Histology images of the biopsy specimen of the Brain. (a) Hematoxylin and eosin photomicrograph high power view (400x) of high-grade small ovoid poorly differentiated neoplastic cells with a high N:C ratio (curved arrows) in the brain parenchyma (straight arrow). (b) Hematoxylin and eosin photomicrograph low power view (100x) of high-grade small ovoid poorly differentiated neoplastic cells with a high N:C ratio (curved arrow) with scant areas of the brain parenchyma (straight arrows).

On day eighteen, the patient had a right-sided open resection craniotomy with biopsy and left-sided stereotactic cyst drainage. The pathology of the specimen showed high-grade, nonlymphoid neoplasm favoring glioblastoma with undifferentiated small cell components (Figure 2) with O-methylguanine-DNA-methyltransferase (MGMT) promoter methylated. Next-generation sequencing with glioma panel detected mutation in PTEN and APC with wild-type IDH1 and wild-type IDH2. The patient was deemed unfit for surgery and was initially offered enrollment in clinical trial at Memorial Sloan Kettering Cancer Center; however, he was not interested. The patient ultimately elected to be treated with the standard of care of concurrent chemotherapy with radiotherapy followed by adjuvant chemotherapy with temozolomide based on the Stupp trial [3]. His treatment was modified to a shorter course of radiotherapy with concurrent temozolomide followed by adjuvant chemotherapy based on the study by Perry et al. [4]. The patient received a total dose of 40.05 Gy, given in 15 daily fractions over three weeks, along with concurrent temozolomide (75 mg/m^2^) for twenty-one days. Interval CT of the head without contrast 10 days after initial treatment showed decreased size of the brain lesions. The patient did not receive adjuvant temozolomide (150-200 mg/m^2^) for five consecutive days of a twenty-eight-day cycle for up to 12 cycles as he decided to return to Haiti.

## 3. Discussion

Glioblastoma was first described by Dr. Bradley W. L in 1880 [18]. It has been identified as a tumor with a median survival of only 15–16 months and a 5-year survival rate of 5–10% [19]. GBM makes up about eighty percent of all primary tumors of the brain [20]. A thorough literature review was conducted on PubMed using the following keywords: “cystic glioblastoma”, “differential diagnosis of cystic glioblastoma”, “presentation of cystic glioblastoma”, and “treatment of cystic glioblastoma”.

Table 1 shows a comprehensive literature review of the common characteristics of cystic glioblastoma. There were nine GBM cases with cystic components. Of those nine cases, six were men, and three were women, corresponding to 67% and 33%, which is similar to the incidence rate in all GBM reported in the literature [21]. Also, the typical age group for the presentation of GBM is between fifty-five and sixty years though it can occur at any age [22]. In the nine cases reported in Table 1, 45% were less than 55 years, 33% were between 55 and 70 years, and 22% were more than 70 years.

The most common signs and symptoms in patients with cystic GBM seen in Table 1 are difficulties of speech (45%), headache (33%), seizure (33%), hemiparesis (22%), and peripheral vision disturbance (11%) which is similar to all GBM from the literature [23]. In addition, some patients have psychiatric symptoms, dermatological hypoesthesia, and vertigo [24]. In our case, the patient had progressive right-sided weakness and dysesthesias.

Diagnosing cystic glioblastoma can be challenging with broad range of differential diagnosis including bacterial abscess, tuberculosis, parasitic infections such as neurocysticercosis, sarcoidosis, and primary and secondary brain tumors [25]. Although rare, infectious processes can mimic GBM as noted in Table 2. In our case, the patient was initially treated for neurocysticercosis; however, there was still a growing concern for malignancy. While GBM generally presents as a solitary solid lesion, however, GBM presenting as a cystic lesion is a rare occurrence [26]. GBM presenting as multiple cystic lesions is rarer as reported by Kumar et al. [7].

Magnetic resonance imaging (MRI) is the gold standard modality for imaging as it can better show the complexity and the heterogeneity of the tumor lesions compared to a CT scan [27]. Surgical resection and biopsies are the gold standards for confirming the diagnosis of glioblastoma [28]. All the cases in Table 1 required tissue to confirm the diagnosis of GBM. Our case was challenging as the brain lesions mimicked an infectious process and a biopsy was needed to confirm the diagnosis of GBM after the seventeenth day of admission.

The most common mutations involving GBM are TP53, TERT, and PTEN, including amplifications in EFGR, PDGFRA, CDK4, CDK6, MDM2, and MDM4. IDH1 and IDH2 mutations along with methylation of the MGMT have been associated with a favorable prognosis. Numerous studies have shown that MGMT methylation was associated with improved overall survival in patients receiving temozolomide and radiotherapy or radiotherapy alone [29, 30]. Median survival for patients with methylation versus lack of methylation is 21 months versus 15 months. Mutations in EGFR, CDK4, and MDM2 confer a poor prognosis [26].

The median survival of GBM is generally poor, with a median survival of less than a year [31]. While the literature frequently outlines the treatment of GBM with solid lesions, however, there are few case reports describing the treatment of GBM with cystic lesions. The standard of care for the treatment of GBM includes surgical resection, radiation, and chemotherapy; however, interdisciplinary discussion and clinical trials are often encouraged. Surgical resection is often difficult due to the procedure's invasive nature and the tumor's aggressiveness involving key areas of the brain whose resection could lead to poor quality of life and would not cure the condition [3]. In our case, our patient had advanced disease and was not a suitable candidate for any operative procedure because of the widespread distribution of his lesions.

Besides the use of temozolomide, there are clinical trials using anti-VEGF therapy, a vaccine against EGFRvIII and MGMT inhibitors. Cloughesy et al. added bevacizumab to the current standard of care, which improved progression-free survival (PFS) in a patient with GBM [32]. Other antiangiogenic drugs in clinical trials include cediranib and cilengitide [33, 34]. Another modality explored by Sampson et al. is the use of a vaccine against epidermal growth factor variant III (EGFRvIII) which showed a median survival of 22 months [35]. A study by Adair et al. utilized a novel gene therapy using mutant methylguanine methyltransferase gene-modified hematopoietic stem and progenitor cells to mitigate myelosuppression by alkylating agents in three glioblastoma patients, of which one patient had PFS of more than 2 years after diagnosis. The longest surviving patient in their study is currently more than 40 months from initial diagnosis. Reported side effects from this trial were mild and reversible [36].

In our case, the patient was treated with a short course of concurrent radiotherapy with temozolomide [4], showing response to treatment with decreasing size of his brain lesion. However, he was unable to complete his adjuvant chemotherapy as he decided to return to his home country.

## 4. Conclusion

Cystic glioblastoma can present in unusual ways, most commonly as a mimicker of infection, which can delay diagnosis and appropriate therapy. This case highlights the importance of identifying clinical features and presentations of cystic GBM to ensure early diagnosis and treatment. Clinicians need to maintain vigilance when encountering a patient with any lesions in the brain and keep GBM as part of the differential. An aggressive approach with a biopsy might be beneficial when the initial workup for a cystic lesion is unclear. It is also clear that glioblastoma, whether cystic or noncystic, is treated in one of two ways. The standard of care includes surgery, radiotherapy with concomitant temozolomide, or participation in a clinical trial. With limited treatment options and poor clinical outcome, the treatment decision is ultimately a shared decision between the patient and the clinician. There are new promising therapies; however, further clinical investigation is warranted for such devastating disease.

## Figures and Tables

**Table 1:** Literature search in PubMed using the following keywords: “cystic glioblastoma”, “differential diagnosis of cystic glioblastoma”, “presentation of cystic glioblastoma”, and “treatment of cystic glioblastoma”.

Article title	Age (in years) and sex of patient	Initial symptoms	Presentation on imaging	Procedure	Treatment	Ref. no.
“Cystic Glioblastoma Rupturing into the Ventricle” PMID: 31938681	77 males	Disorientation and left facial weakness.	A neoplastic lesion with a large cystic structure at his right frontal lobe.	Surgical resection on day 10	TMZ maintenance. Recurrence treated with gamma knife. Bevacizumab was added to the TMZ maintenance.	[5]
“Cerebellar Cystic Glioblastoma: An Uncommon Presentation of a Rare Disease and Clinical Review” PMID: 30623119	64 females	A 2-week history of dysarthria and dysphagia.	A hypodense lesion in the posterior fossa with intralesional hemorrhage.	Surgical resection of tumor two months after initial admission	Concurrent chemotherapy with radiotherapy followed by adjuvant chemotherapy with temozolomide.	[6]
“Bilateral Cystic Glioblastoma Multiforme” PMID: 24347967	83 females	Right hemiparesis and inability to speak for 4 days.	Large enhancing cystic lesions measuring 4 × 2.8 × 3 cm and 4.5 × 4 × 3.4 cm in the right and left frontal regions.	Biopsy of cyst wall in a few days of admission	The prognosis was explained. The patient did not consent to definitive surgery. She died 5 months after discharge.	[7]
“Atypical Presentation of Glioblastoma Multiforme” PMID: 30756069	53 females	A 2-week history of lack of coordination in her hands and some difficulty in speech.	Atypical neurological presentation.	Stereotactic surgical resection within 2 days	Not reported.	[8]
“Cystic Glioblastoma Multiforme Masquerading as a Cerebral Tuberculoma” PMID: 25326570	45 males	Seizures and headache.	Mass in the right temporoparietal region with a hypodense center surrounded by a ring of enhancement.	Surgical resection and histopathological examination	Surgery was repeated for maximum debulking of the tumor.	[9]
“Glioblastoma Masquerading as Herpes Simplex Encephalitis” PMID: 25443666	50 males	Headache, confusion, and an episode of seizure.	A hypodense lesion in the left frontoparietal region with CSF analysis showing positive herpes simplex virus.	Lumbar puncture	Acyclovir and prednisone initially followed by surgical resection.	[10]
“Rapid Progression of Glioblastoma Multiforme: A Case Report” PMID: 28105188	60 males	Altered mental status after seizure.	Multiple lesions between the left temporal and left occipital lobes, which had increased in size.	Lumbar puncture	On initial admission with mannitol and antiseizure medication along with antiparasitic treatment when GBM was found.	[11]
“Transtentorial Spread of Glioblastoma Multiforme to Cerebellopontine Angle – A Rare Case Report” PMID: 35127205	55 males	2-month history of progressive left frontal headache associated with nausea, vomiting, and an episode of confusion.	Mass evolving in the left parietal and occipital lobes, measuring 4.9 × 1.7 × 3.7 cm, and compressing the ipsilateral ventricle.	Surgical resection	The patient underwent adjuvant treatment with 60 Gy focal brain radiotherapy fractioned in 30 days and 12 cycles of temozolomide.	[12]
“Large Cystic Glioblastoma Multiforme” PMID: 22286148	41 males	Dizziness and transient right hemiparesis.	5 cm sized cystic mass in the left parietal lobe.	Total surgical resection	Fractionated stereotactic radiotherapy (FSRT) along with 3 cycles of chemotherapy including nimustine (ACNU) and cisplatin given for 3 days and additionally 4 cycles of temozolomide given for 5 days every 1 month.	[13]

**Table 2:** Noncancerous pathology mimicking glioblastoma.

Article title	Age (in years) and sex of patient	Initial symptoms	Presentation on imaging	Procedure	Treatment	Ref. no.
“Cerebral Cryptococcoma Mimicking Glioblastoma” PMID: 28188169	55 males	1-month history of headaches	Encapsulated cryptococcoma presenting as a large cystic lesion mistaken for GBM	Lumbar puncture with CSF examination	Dexamethasone, amphotericin, and 5-flucytosine initially followed by surgical resection	[14]
“Intracerebral Neurocysticercosis Mimicking Glioblastoma Multiforme: A Rare Differential Diagnosis in Central Europe” PMID: 11305755	47 males	A 4-week history of speech difficulties	Low-density multilobulated cystic frontal mass with peripheral ring contrast enhancement adjacent to the Sylvian fissure	Surgical removal and histologic examination	Surgical resection	[15]
“Basal Ganglia Infarction Mimicking Glioblastoma” PMID: 16183553	52 males	Acute brachiofacial paresis	A hyperintense lesion with mass effect and ring enhancement in basal ganglia	No biopsy done	Recommended stereotactic brain biopsy, but the patient recovered gradually	[16]
“The Eyes as a Window to the Brain: A Teaching Case Report of Misdiagnosed Glioblastoma”	43 males	Vision loss	Intra-axial oval-shaped mass with a partial fluid component at the left occipital lobe measuring approximately 5 cm long, 7.7 cm anterior-posterior, and 2.7 cm transversely with minimal surrounding edema and some mass effect and midline shift to the left lateral ventricle	Surgical resection	Radiation therapy and oral chemotherapy with temozolomide	[17]

## Data Availability

None is required for this report.
